# Oxytocin induced oxidative stress in lactating *Bubalis bubalis* (Nili Ravi)

**DOI:** 10.1186/1746-6148-9-169

**Published:** 2013-08-27

**Authors:** Zafar Iqbal, Zia Ur Rahman, Faqir Muhammad, Tanweer Khaliq, Haseeb Anwar, Mian Muhammad Awais, Saima Sadaf

**Affiliations:** 1 Department of Basic Sciences, sub campus Jhang, University of Veterinary and Animal Sciences, Lahore, Pakistan; 2 Sub-Campus Toba Tek Singh, University of Agriculture, Faisalabad, Pakistan; 3 Department of Physiology and Pharmacology, University of Agriculture, Faisalabad, Pakistan; 4 Department of Physiology, GC University, Faisalabad, Pakistan; 5 Department of Pathobiology, sub campus Jhang, University of Veterinary and Animal Sciences, Lahore, Pakistan; 6 Government College of Home Economics, Lahore, Pakistan

**Keywords:** Oxytocin, Oxidative stress, Biomarkers, Lactating buffaloes

## Abstract

**Background:**

Oxytocin has been reported for a wide range of adverse effects in different species of lactating animals. The present study was aimed to evaluate the adverse effects of oxytocin on biomarkers of oxidative stress in buffaloes. Lactating buffaloes (n = 40) were randomly selected from a commercial dairy farm located in the peri-urban area of district Faisalabad, Pakistan and divided into two equal groups viz. treatment and control groups, each containing 20 buffaloes. Buffaloes in treatment group were injected with oxytocin before each milking (morning and evening) for milk let down; whereas, animals in control group were milked naturally without oxytocin injection. Both the groups were assessed for oxidative stress biomarkers.

**Results:**

Results showed significantly higher levels (P ≤ 0.05) of TOS, tHcy and ceruloplasmin oxidase activity in lactating buffaloes injected with oxytocin as compared to those of control group. On the other hand, serum levels of TAS, PON1 and arylesterase were significantly lower (P ≤ 0.05) in the buffaloes of treatment group.

**Conclusions:**

Oxytocin injection in lactating buffaloes resulted in elevated oxidative stress by increasing the total homocysteine and ceruloplasmin oxidase activity and decreasing enzymatic activities of antioxidant enzymes including paraoxonase-1 and arylesterase; that might render the animals to poor productive and reproductive potential.

## Background

Buffaloes are said to be slow and hard milkers because of their slow milk ejection reflexes and hard teat sphincter muscles [1]. As a general practice, exogenous oxytocin injections are frequently administered in dairy animals to initiate the milk let down or occasionally to cure the disturbed milk ejection [2,3]. However, long term oxytocin administration in dairy animals could lead to addiction and reduced spontaneous milk ejection after withdrawal of oxytocin [4].

Total antioxidant capacity (TAC) provides biologically more relevant information that describes the dynamic equilibrium between pro-oxidants and antioxidants in the plasma compartment of animals and human beings [5]. Under physiological conditions, there are sufficient reserves of antioxidants in the body that has the capability to cope with the production of free radicals [6], which are produced continuously during normal metabolism and may increase as a result of pathological conditions [7]. However, when the production of free radicals exceeds the body’s antioxidant production potential, oxidative stress develops [8]. In dairy animals, pre-partum and early post-partum periods are very critical as they prone the animals to considerable physiological challenges by imposing significant metabolic stressors that may contribute to the onset of diverse disorders including ketosis, milk fever, mineral deficiencies etc. [9]. In Pakistan, oxytocin is widely used in dairy animals to increase the milk production due to the lack of awareness about the health hazards associated with irrational use of oxytocin. Keeping in view, the present study was conducted to determine the effect of oxytocin injection on health/oxidative stress biomarkers of lactating buffaloes.

## Results

### Indicators of oxidative stress

#### Total oxidant status (TOS) and total antioxidant capacity (TAC)

In oxytocin injected group, the serum TOS (μMol/L) was significantly higher (P ≤ 0.05) during 1st (0.97) and 5th month (0.98) of lactation as compared to control group (not injected with oxytocin). TAC (mMol/L) was significantly higher (P ≤ 0.05) in control buffaloes on 2nd (0.40), 3rd (1.06), 4th (0.78) and 6th months (0.55) of lactation as compared to oxytocin injected buffaloes; whereas, the highest value for TAC was recorded on 3rd month (1.06) of lactation in control group (Table 1). Pearson correlation coefficients indicated that TOS was positively correlated (r = 0.604) with TAC in the control buffaloes (Table 2); whereas, in treated buffaloes no such significance was detected.

**Table 1:** **Oxidative stress parameters (Mean ± SE) of control and treated (oxytocin injected) lactating *Bubalis bubalis *(Nili Ravi) during different months of lactation**

**Parameters**	**1st month**		**2nd month**		**3rd month**		**4th month**		**5th month**		**6th month**	
	**C**	**T**	**C**	**T**	**C**	**T**	**C**	**T**	**C**	**T**	**C**	**T**
**TOS (μMol/L)**	0.47 ± 0.02^c^	0.97 ± 0.08^ab^	0.79 ± 0.09^b^	0.79 ± 0.09^b^	1.10 ± 0.07^a^	1.12 ± 0.08^a^	1.08 ± 0.06^a^	1.00 ± 0.05^ab^	0.36 ± 0.03^c^	0.98 ± 0.071^ab^	0.78 ± 0.09^b^	0.97 ± 0.06^ab^
**TAC (mMol/L)**	0.30 ± 0.01^efg^	0.20 ± 0.02^fg^	0.40 ± 0.06^de^	0.26 ± 0.02^fg^	1.06 ± 0.02^a^	0.49 ± 0.06^cd^	0.78 ± 0.03^b^	0.32 ± 0.02^ef^	0.18 ± 0.02^g^	0.28 ± 0.02^efg^	0.55 ± 0.08^c^	0.25 ± 0.01^fg^
**tHcy (μMol/L)**	12.72 ± 0.59^cd^	16.77 ± 0.99^a^	10.43 ± 0.30^d^	17.04 ± 0.84^a^	14.20 ± 0.65^bc^	15.77 ± 0.66^ab^	12.15 ± 0.86^cd^	17.52 ± 0.57^a^	14.03 ± 0.92^bc^	16.52 ± 0.56^ab^	15.32 ± 0.33^ab^	17.44 ± 0.52^a^
**PON1 activity (U/L)**	35.06 ± 2.68^g^	45.74 ± 2.63^bcd^	58.29 ± 2.41^a^	48.71 ± 1.92^bc^	50.41 ± 1.63^bc^	45.59 ± 2.22^bcde^	51.41 ± 2.66^ab^	40.59 ± 1.33^defg^	46.98 ± 1.12^bcd^	38.51 ± 1.46^efg^	43.75 ± 1.23^cdef^	37.64 ± 0.86^fg^
**Arylesterase activity (U/L)**	47.12 ± 1.45^ab^	32.94 ± 1.04^f^	32.98 ± 1.38^ef^	28.09 ± 1.40^f^	43.94 ± 1.59^bc^	45.08 ± 1.75^bc^	38.29 ± 0.80^de^	43.44 ± 2.11^bcd^	40.74 ± 1.35^cd^	30.16 ± 1.35^f^	52.06 ± 0.74^a^	32.86 ± 1.77^f^
**Ceruloplasmin oxidase activity (U/L)**	32.06 ± 2.35^e^	55.91 ± 5.71^bcd^	45.31 ± 2.87^de^	33.09 ± 1.82^e^	51.63 ± 2.09^cd^	50.73 ± 3.80^d^	56.17 ± 4.07^bcd^	100.44 ± 5.54^a^	69.94 ± 4.59^b^	65.20 ± 4.55^bc^	65.31 ± 4.68^bc^	58.16 ± 1.74^bcd^

Values with different alphabets in a row significantly differ at (P ≤ 0.05).

Standard abbreviations and units are given in parenthesis.

“C” = Control group; “T” = Treatment group (oxytocin injected).

“TOS” = Total oxidant status; “TAC” = Total antioxidant capacity.

“tHcy” = Total homocysteine; “PON1” = paraoxonase-1.

**Table 2:** Correlation coefficients of oxidative stress parameters in control lactating (Nili Ravi) buffaloes

**Parameters**	**Total oxidant status**	**Total antioxidant capacity**	**Arylesterase activity**	**Ceruloplasmin oxidase activity**
**Total oxidant status (μMol/L)**		0.604		
		(0.000)		
**Total homocysteine (μMol/L)**	0.481	0.389		
	(0.000)	(0.002)		
**Paraoxonase 1 activity (U/L)**	0.211	0.209	−0.379	0.184
	(0.020)	(0.022)	(0.000)	(0.044)

Values in parenthesis indicate significance (P ≤ 0.05).

Standard abbreviations and units are given in parenthesis.

Blank cells indicate non-significant correlation at P ≥ 0.05.

#### Total homocysteine (tHcy)

A significantly higher (P ≤ 0.05) concentration of tHcy (μMol/L) was recorded in buffaloes of oxytocin injected group on 1st (16.77), 2nd (17.04) and 4th months (17.52) of lactation when compared with those of control group; whereas, highest tHcy concentration was detected on 4th month (17.52) of lactation (Table 1). Correlation analysis revealed a positive correlation of tHcy with TOS (r = 0.481) and TAC (r = 0.389; Table 2).

#### Paraoxonase-1 (PON1) activity

Buffaloes of control group showed significantly higher (P ≤ 0.05) serum PON1 activity (U/L) during 2nd (58.29), 4th (51.41) and 5th months (46.98) of lactation as compared to those of oxytocin injected group which showed significantly higher PON1 activity during 1st month (45.74) of lactation (Table 1). PON1 activity of control group was positively correlated with TOS (r = 0.211), TAC (r = 0.209), ceruloplasmin oxidase activity (r = 0.184) and negatively correlated with arylesterase activity (r = –0.379; Table 2). PON1 activity of the lactating buffaloes injected with oxytocin showed a negative correlation with ceruloplasmin oxidase activity (r = −0.255).

#### Arylesterase activity

The serum arylesterase activity (U/L) increased significantly (P ≤ 0.05) during 1st (47.12), 5th (40.74) and 6th months (52.06) of lactation and the highest level was observed during 6th month (52.06) in the control group (Table 1). Arylesterase activity of oxytocin injected group was positively correlated with TAC (r = 0.324) and ceruloplasmin oxidase activity (r = 0.199).

#### Ceruloplasmin oxidase activity

Animals injected with oxytocin showed significantly higher (P ≤ 0.05) ceruloplasmin oxidase activity (U/L) during 1st (55.91) and 4th months (100.44) of lactation as compared to control group; whereas, on 3rd month of lactation the difference between the ceruloplasmin oxidase activity of both the groups was statistically similar (P ≥ 0.05) (Table 1).

## Discussion

Oxytocin has been reported for a wide range of adverse effects in lactating animals including sheep, goat, cattle and buffaloes [2-4]. In lactating animals, the start of lactation is an important event with respect to the production of free radical(s). It has been reported in the literature that in malnourished lactating animals, negative energy balance is developed and in such circumstances, oxytocin favors the production of free radicals through mobilization of stored lipids and gluconeogenesis [10,11]. Further, excessive oxidation of non-esterified fatty acids (NEFA) in the liver also increased the production of reactive oxygen species (ROS) which ultimately produced the oxidative stress [12].

In the present study, statistically TOS was higher and TAC was lower in oxytocin injected buffaloes and these results are consistent to the findings of [6] who conducted similar studies in lactating cows. This might be due to the depletion of fat soluble antioxidants by the milk [8,13]. Further, this reduction might also be due to decreased level of PON1 [14] that was another peculiar finding of this study. Moreover, in some previous studies, reduction in TAC just after calving had also been correlated with increased production of reactive oxygen metabolites (ROM) and thiobarbituric acid reactive substance. Elevation in these reactive substances induced imbalance between TAC and production of free radicals leading to lipid peroxidation [15].

Oxidative stress has an important role in the metabolism of homocysteine. In present study, significantly higher tHcy level was detected in oxytocin injected buffaloes that might be correlated with higher level of free radicals in circulation [16]. Similarly, [10] also observed a significant increase in the serum homocysteine on day 200 of lactation in the lactating ewes. Altered plasma homocysteine levels had also been correlated with certain dietary factors such as protein and vitamin deficiencies [16,17], renal dysfunction and hypothyroidism [18].

The results of enzymatic activities revealed significantly lower PON1 and arylesterase levels in oxytocin injected buffaloes as compared to control [14,19-21] also found a lower PON1 activity in the pregnant, early lactating and late lactating dairy cows. It might be correlated with increased level of homocysteine that triggered the atherosclerosis process due to which expression of PON1 gene in the hepatic tissue was down regulated [22,23]. It could be speculated that proatherogenic effects of tHcy might be involved in the reduction of serum PON1 activity and thus altered antioxidant function. The PON1 enzyme is responsible for both paraoxonase and arylesterase activities in bovine serum, because it hydrolyses organophosphates (such as paraoxon) and aromatic esters such as phenyl acetate [24,25]. Moreover, the elevated oxidative stress in the form of free radicals in the present study could lower PON1 activity [14,19]; whereas, increased lipid metabolism in the form of lipid peroxidation might also result in lower PON1 activity [21]. The lipids act as substrate for lipid peroxidation and an inverse relationship exists between lipid peroxidation and PON1 activity [26]. Additionally, inflammatory conditions induced by oxytocin injection could also be responsible for elevated oxidative stress, which directly decreased the PON1 activity [27].

Ceruloplasmin oxidase activity was found significantly higher in oxytocin injected buffaloes as compared to control. Ceruloplasmin has oxidase activity and is related with the host acute phase responses [28] and antioxidant defense in cattle [29,30] so, its level might be enhanced during inflammation, tissue insult, certain malignant tumors [31,32] and oxidative stress to quench the free radicals, produced during oxidative stress. So, ceruloplasmin oxidase activity is the important health parameter that could be used to judge the health status and well-being of the animals [33]. Previous studies reported that higher ceruloplasmin oxidase activity in freshly calved animals is linked with a physiological phenomenon [34-36]. In the present study, increased activity of ceruloplasmin might be due to pathological inflammatory condition, induced by oxytocin [27]. In inflammatory conditions, cytokines such as interleukin-1 and interleukin-6 are released from leukocytes which stimulate the liver to secrete ceruloplasmin [37].

## Conclusions

In conclusion, oxytocin injection in lactating animals resulted in higher oxidative stress by enhancing the total homocysteine level and ceruloplasmin oxidase activity and decreasing enzymatic activities of antioxidant enzymes including paraoxonase-1 and arylesterase. The results of present study discourage the irrational use of oxytocin for milk let down in lactating animals that may prone the animals to poor productive performance and reproductive efficiency.

## Methods

### Experimental design

Healthy, 4-7 years old freshly calved lactating (Nili Ravi) buffaloes (n = 40) were randomly selected and maintained on a commercial dairy farm located in the peri-urban area of district Faisalabad, Pakistan. The animals were allocated into two equal groups (n = 20, each) viz. treatment (oxytocin injected) and control (without oxytocin injection) groups.

Both the groups were assigned to 4 replicates, 5 animals each. All the animals were milked twice a day (4.00 am and 4.00 pm), by injecting 3 ml of oxytocin (10 IU/mL; Star Laboratories, Pakistan) 3–5 minutes before each milking.

Blood sampling was started exactly from day 15th post delivery that continued for a period of six months with an interval of 15 days between two successive samplings. Blood samples were collected from jugular vein using BD-vacutainer and sera samples were separated in eppendorf tubes from collected blood samples at 1107×g for 15 minutes and stored at −20°C till analysis. All animal care and experimental procedures were carried out in accordance with the guide for the humane use and care of animals, approved by the animal care committee of University of Agriculture-Faisalabad, Pakistan.

### Determination of oxidative stress

#### Measurement of total oxidant status (TOS)

The TOS of the body was estimated using the method, developed by [38]. Briefly, 35 μl of serum samples were mixed with 225 μL of reagent 1 (150 μM xylenol orange, 140 mM NaCl and 1.35 M glycerol having a total volume of 1000 ml; pH, 1.75). After mixing, the first absorbance was read. Then, 11 μL of reagent 2 (5 mM ferrous ammonium sulfate and 10 mM O-dianisidine dihydrochloride) was mixed with the solution containing reagent 1 and the serum sample. The final absorbance was read after 4 minutes after mixing with reagent 2 at bichromatic wavelengths, using a main wavelength of 560 nm and secondary/differential wavelength of 800 nm. Then the TOS concentrations were calculated in terms of micromolar per liter of hydrogen peroxide.

#### Measurement of total antioxidant capacity (TAC)

The total antioxidant capacity of the body was determined by using a novel automated ABTS radical cation method [39]. Briefly, 5 μl of serum samples were mixed with 200 μl of reagent 1 (0.4 M/L sodium acetate and 0.4 M/L glacial acetic acid; pH 5.8). Then 20 μl of reagent 2 (30 mM/L acetate buffer solution, 2 mM/L H2O2 and 10 mM/L 2,2′-azinobis 3-ethylbenzothiazoline-6-sulfonate; ABTS) was added to this mixture and reading was taken after 5 minutes of incubation at 37°C. This absorbance was used to determine the TAC in terms of milimolar per liter of Trolox (Sigma, Vit. E analogue).

#### Total homocysteine

The quantitative determination of total L-homocysteine in the serum was determined by using the commercially available kit (The Diazyme Homocysteine Enzymatic Assay Kit; Ref. # DZ 568A-K; Diazyme Laboratories, Gregg Court Poway, CA 92,064, USA).

### Determination of enzymatic activities

#### Paraoxonase-1(PON1) activity

The PON1 activity was determined by the hydrolysis of paraoxone into p-nitrophenol as described by [40]. Briefly, 10 μL of serum sample was mixed with 350 μL of paraoxonase substrate reagent 0.1 M Tris–HCl buffer of pH 8.0, 2 mM paraoxone (Sigma-Aldrich Laborchemi Kalien GmbHD-30,918, Seelze, Germany) as substrate and 2 mM CaCl_2_ without NaCl stimulation. The generation of p-nitrophenol was monitored at 405 nm on spectrophotometer Biosystem, BTS-330 (Biosystems, S.A. Costa Brava, Barcelona, Spain). The PON1 activity in terms of international units (U/L) was calculated by using the formula; [PON1 activity(U/L) = (Absorbance/0.017) × 50]

Wheres, 50 = dilution factor

0.017 = Micromolar absorptivity of the p-nitrophenol.

### Arylesterase activity

Arylesterase activity was measured in term of phenylacetate hydrolysis as described by [41]. The reaction mixture contained 350 μL of arylesterase substrate (2.0 mM phenylacetate, 2 mM CaCl_2_ in 0.1 M Tris–HCl buffer of pH 8.0) and 10 μL of serum. The initial rate of hydrolysis was determined following the increase in arylesterase activity at 270 nm (Biosystem, BTS-330). The enzyme activity (U/L) was calculated by using the same formula as mentioned for PON1 Activity.

### Ceruloplasmin oxidase activity

The enzymatic activity of ceruloplasmin oxidase was measured by using the slightly modified colorimetric method of [42]. Briefly, 750 μl of acetate buffer (pH 5) was mixed with 50 μl of the serum sample and incubated at 30°C (water bath) in duplicate test tubes for 5 minutes. Then 200 μl of substrate (250 mg of ortho-dianisidine dihydrochloride (Sigma) was brought upto 100 ml with deionized water), was added to both the test tubes. After 5 and 15 minutes of substrate addition, 2 ml of sulfuric acid (9 M) was added to stop the reaction. The absorbance of both the test tubes was taken at 540 nm wavelength by using a UV/Vis spectrophotometer (Hitachi U-2001, USA). Then following formula was used to calculate the enzymatic activity.

$$\mathrm{Ceruloplasmin}\;\mathrm{oxidase}\;\mathrm{activity}\;\left(U/L\right)={\mathrm{Abs}}_{15\min}{‒\mathrm{Abs}}_{5\min}\times 6.25\;\times 10^{2}$$

Whereas,

Abs_15min_ and Abs_5min_ were the absorbance of samples after 15 and 5 minutes respectively. 6.25 × 10^2^ was the dilution factor of the reaction mixture.

### Statistical analysis

Data were analyzed statistically using two way analysis of variance, whereas, the means for significance between different groups was detected by using Duncan Multiple Range Test (DMR). All the values were considered significant at P ≤ 0.05.

## Competing interests

The authors declare that they have no competing interests.

## Authors’ contributions

ZI and ZUR designed and conducted the study. ZI and MMA wrote the manuscript and analyzed the data. HA, and SS performed the analysis. FM and TK helped to write the manuscript. All authors have read and approved the final manuscript.
